# Investigating the Role of Gut Microbiota in Pediatric Patients with Severe COVID-19 or MIS-C

**DOI:** 10.3390/microorganisms13010083

**Published:** 2025-01-04

**Authors:** Elena Franchitti, Paolo Bottino, Francesca Sidoti, Andrea Carpino, Giulia Pruccoli, Ugo Ramenghi, Cristina Costa, Ugo Ala, Emilia Parodi, Deborah Traversi

**Affiliations:** 1 Department of Public Health and Paediatrics, University of Turin, Piazza Polonia 94, 10126 Torino, Italy; elena.franchitti@unito.it (E.F.); paolo.bottino@unito.it (P.B.); ugo.ramenghi@unito.it (U.R.); cristina.costa@unito.it (C.C.); 2 Microbiology and Virology Laboratory, University Hospital “SS Antonio e Biagio e C. Arrigo”, Via Venezia 8 16, 15121 Alessandria, Italy; 3 Division of Virology, Azienda Ospedaliero Universitaria—Città Della Salute e Della Scienza, Corso Bramante 88, 10126 Torino, Italy; francesca.sidoti@unito.it; 4 Regina Margherita Paediartic Hospital, Azienda Ospedaliero Universitaria—Città Della Salute e Della Scienza, Piazza Polonia 94, 10126 Torino, Italygiulia.pruccoli@unito.it (G.P.); 5 Department of Veterinary Science, University of Turin, Largo Paolo Braccini 2, 10095 Grugliasco, Italy; ugo.ala@unito.it; 6 Struttura Complessa Pediatrica e Neonatologia, Azienda Ospedaliera Ordine Mauriziano Via Magellano 1, 10128 Torino, Italy; emilia.parodi@unito.it

**Keywords:** SARS-CoV-2, COVID-19, MIS-C, children, gut microbiota, qRT-PCR, *16S rRNA*, NGS, *Akkermansia muciniphila*, *Bifidobacterium* spp.

## Abstract

Severe COVID-19 and MIS-C are rare but serious outcomes associated with SARS-CoV-2 infection. The onset of MIS-C often involves the gastrointestinal system, suggesting a potential connection with gut microbiota. This study aims to compare the gut microbiota of children with severe COVID-19 and those with MIS-C using various biomolecular approaches. Gut microbiota composition and specific microbial modulations were analyzed using fecal samples collected at hospital admission. The study included hospitalized patients (mean age 6 ± 5 years) diagnosed with severe COVID-19 (37 patients) or MIS-C (37 patients). Microbial differences were assessed using both NGS and qRT-PCR methodologies. In 75% of cases, pharmacological treatments included antibiotics and corticosteroids, which influenced the microbiota composition. Early age was found to have the most significant impact on microbiota diversity. Significant differences in alpha and beta diversity were observed between COVID-19 and MIS-C patients, particularly concerning low-abundance species. Levels of *Bacteroides* spp., *Bifidobacterium* spp., and *Akkermansia muciniphila* were comparable between groups, while an increased activity of *Bifidobacterium* spp. was noted in children with positive fecal samples (*p* = 0.019). An in-depth evaluation of lesser-known gut species may be key to reducing the risk of severe outcomes and developing microbiota-based biomarkers for the early diagnosis of MIS-C.

## 1. Introduction

Gastrointestinal symptoms are common manifestations of SARS-CoV-2 infection [1], and SARS-CoV-2 RNA can be detected in the stool of 30–40% of infected individuals. The infectivity of the virus in the gut remains largely unknown, although preliminary evidence suggests the presence of a non-pathogenic virus in stool samples [2]. A higher expression of ACE2 in the gut of children compared to lower levels in the respiratory tract [3] is associated with a greater percentage of positive stool samples (up to 80%), a higher frequency of gastrointestinal symptoms [4], and the predominance of asymptomatic, paucisymptomatic, or mild forms of COVID-19 in this population [5]. In Italy, to date, surveillance has reported approximately 5 million cases among individuals aged 0–19 since the beginning of 2020, of which 0.5% required hospitalization [6], primarily affecting infants under 1 year of age.

Multisystem inflammatory syndrome (MIS) is a rare but severe condition that can manifest in adults (MIS-A) and children (MIS-C) 2–6 weeks after suspected exposure [7]. MIS is characterized by a pro-inflammatory state leading to multi-organ failure, primarily affecting the cardiovascular, gastrointestinal, and neurological systems [8]. Recently, autoimmune mechanisms have been proposed as a potential contributor to MIS-C pathogenesis [9]. The global incidence of MIS-C is estimated at 3 cases per 10,000 infections, with a higher prevalence observed in children aged 8–11 years [10].

In the United States, the overall incidence of MIS-C during 2023 was 0.11 cases per million person-months (95% CI = 0.10–0.14), representing a 98% decline compared to the peak incidence of 6.79 (95% CI = 6.56–7.03) observed early in the COVID-19 pandemic (October 2020–April 2021) [11]. Despite the significant reduction in incidence, MIS-C remains a severe delayed hyperinflammatory condition in children and adolescents. Its pathogenesis is still not fully understood, with an estimated mortality rate of 1–2% [12].

In all clinical conditions, the presence of SARS-CoV-2 in the gut increases intestinal mucosal permeability, leading to mild dysbiosis [13,14]. Microbiota alterations include a reduction in both alpha diversity and short-chain fatty acid producers, along with an increase in pathogenic microorganisms [15].

Although several clinical studies have been conducted on limited cohorts, focusing on accurate diagnosis [16,17] and treatment efficacy [18], further mechanistic research is needed to clarify the still-unknown pathological pathways [18]. The involvement of the gut microbiome in the progression of MIS-C remains unclear, primarily due to the small cohort sizes and differing assessment methodologies. However, a distinct microbial signature has been identified in pediatric patients, with a noticeable influence from preventative antibiotic treatments, which were especially common during the early phase of the pandemic [15]. Notably, only minor differences in gut microbiota involving commonly studied microorganisms, such as *Bifidobacterium bifidum* and *Akkermansia muciniphila*, have been observed in children as a consequence of SARS-CoV-2 infection [19,20].

This study aims to compare the gut microbiome and specific microbial targets in children affected by COVID-19 or MIS-C in Northern Italy, with the goal of developing early gut-based biomarkers for MIS-C onset.

## 2. Materials and Methods

### 2.1. Study Design and Sample Collection

During the pandemic, between 15 April 2020 and 29 February 2022, a total of 74 stool samples were collected at the Regina Margherita Children’s Hospital, the main pediatric hub in Piedmont. Among these, 37 samples were obtained from MIS-C patients and 37 from children with COVID-19. Verbal informed consent was obtained from the parents or legal guardians of all participants, and a questionnaire was completed to gather clinical and demographic data.

Each enrolled patient underwent a nasopharyngeal swab and a serological test to detect anti-SARS-CoV-2 antibodies using an enzyme-linked immunosorbent assay (ELISA) [21]. Demographic information, clinical presentation, and the time interval from SARS-CoV-2 infection to stool sample collection were recorded in the hospital’s digital database.

All enrolled children were unvaccinated against COVID-19, as vaccination for pediatric populations was not yet available at the time. Stool samples were collected during the first evacuation following hospital admission, stored at −80 °C, and subsequently transported to the Hygiene Laboratory within the Department of Public Health and Pediatrics for analysis. Given the challenges of stool sample collection in children compared to adults, all samples were carefully assessed and categorized as types 3 to 5 on the Bristol Stool Chart.

### 2.2. Nucleic Acids Extraction and Quantification

Both DNA and RNA were extracted from the fecal samples. Total nucleic acid extraction was performed using the PowerFecal DNA and PowerMicrobiome RNA isolation kits (QIAGEN, Venlo, The Netherlands). Quantification of the extracted nucleic acids was carried out with a Tecan Infinite^®^ 200 PRO spectrophotometer, utilizing a NanoQuant Plate (Tecan Trading AG, Männedorf, Switzerland) and the iControl™ software (version 1.11.10), which enables spectrophotometric readings at 260 nm (Table 1).

The 260/280 and 260/230 absorbance ratios were satisfactory across all extracts, indicating good-quality nucleic acids. The DNA integrity number (DIN) of the extracted genomic DNA, as assessed using a TapeStation 4150 (Agilent Technologies, Santa Clara, CA, USA), averaged 6.45 ± 0.55, with no significant differences observed between the MIS-C and COVID-19 samples. The DNA samples were stored at −20 °C, while the RNA samples were stored at −80 °C until molecular analyses were conducted.

### 2.3. SARS-CoV-2 Stool Positivity

RNA extracts were tested for SARS-CoV-2 positivity using the Novel Coronavirus (2019-nCoV) Real-Time Multiplex RT-PCR Kit (LifeRiver Ltd., Shanghai, China) on CFX Instruments (Bio-Rad, Hercules, CA, USA). The analysis followed the thermal protocol provided in the kit instructions and as recommended in the literature [22]. This assay simultaneously targets three viral genes: gene *E*, gene *N*, and gene *RdRp*.

Gene *E* encodes the Envelope small membrane protein, which exhibits ion channel activity and plays a role in the formation of the viral envelope membrane, inducing cell membrane curvature and scission. Gene *N* encodes the Nucleoprotein, which packages the positive-strand viral genome into a helical ribonucleocapsid (RNP) and connects the viral genome to the membrane by interacting with the M protein. Gene *RdRp* encodes the RNA-dependent RNA polymerase, an enzyme essential for viral genome replication. A sample was considered positive if at least two out of the three target genes were detected before the 41st PCR cycle.

### 2.4. Next-Generation Sequencing

Microbiota analysis was performed, starting with the extraction and purification of bacterial DNA. DNA extracts were prepared for next-generation sequencing (NGS) analysis of the *16S rRNA* gene by constructing metagenomic libraries, each uniquely barcoded. The microbial composition was analyzed for each sample using the Ion 16S™ Metagenomics Kit (Thermo Fisher Scientific, Waltham, MA, USA), following the manufacturer’s instructions.

Briefly, an initial multiplex PCR assay was conducted to amplify hypervariable regions of the *16S rRNA* gene (V2, V4, and V8 in one tube, and V3, V6–V7, and V9 in another). The first reaction produced amplicons of approximately 250 bp, 288 bp, and 295 bp, while the second generated fragments of approximately 215 bp, 260 bp, and 209 bp using primers and reagents supplied with the kit. The amplified products were pooled in equimolar quantities and purified using the Agencourt Ampure reagent (Life Technologies, Carlsbad, CA, USA). The DNA concentration was measured with the Qubit 4 Fluorometer (Thermo Fisher Scientific, Waltham, MA, USA) according to the manufacturer’s protocol.

Subsequent steps included end-repair of the fragments and ligation of adapters and barcodes using the Ion Xpress™ Barcode Adapters 1–16 Kit (Thermo Fisher Scientific, Waltham, MA, USA). A secondary PCR amplification of the barcode-ligated library was performed using the Ion Plus Fragment Library Kit (Thermo Fisher Scientific, Waltham, MA, USA). Libraries were quantified by qPCR using the Ion Universal Library Quantitation Kit (Thermo Fisher Scientific, Waltham, MA, USA), diluted to equimolar concentrations, and pooled into a metagenomic library.

NGS of the prepared library was performed following emulsion PCR and chip preparation using the Ion Chef™ system (Thermo Fisher Scientific, Waltham, MA, USA). Sequencing was carried out on the Ion S5 system using an Ion 530™ Chip (Thermo Fisher Scientific, Waltham, MA, USA) according to the manufacturer’s instructions.

The resulting reads were aligned and analyzed using Ion Reporter™ software with default parameters (Thermo Fisher Scientific, Waltham, MA, USA). This platform provided comprehensive metagenomic analysis, including read mapping, annotation, and reporting.

### 2.5. Microbiota Biomarkers Analysis

RNA extracts were converted into complementary DNA (cDNA) using the iScript cDNA Synthesis Kit (Hercules, CA, USA). Two microliters of the RNA extract were processed according to the manufacturer’s instructions. The resulting 20 µL of cDNA was immediately diluted to a final volume of 60 µL and stored at −20 °C.

Quantitative real-time PCR (qPCR) assays were then performed with a CFX Touch System (Bio-Rad, Hercules, CA, USA) for both DNA and cDNA samples to quantify the following targets: Bacteroidetes, Firmicutes, *Bacteroides* spp., *Bifidobacterium* spp., and *Akkermansia muciniphila*. Primers targeting the 16S region of the 30S subunit of the rRNA gene were used, as this region contains hypervariable regions (V1–V9), which are suitable for taxonomic identification. Standard curves were generated from six serial dilutions of certified genomic DNA. The primers and genomic DNA used are detailed in a previous study [23]. Negative controls (without DNA) were performed using ultrapure water. Each PCR assay was conducted in triplicate.

For the targets Bacteroidetes, Firmicutes, *Bacteroides* spp., *Bifidobacterium* spp., and *Akkermansia muciniphila*, 2 µL of extracted DNA or cDNA (pure or diluted) was added to a reaction mix containing 10 µL of SsoAdvanced™ Universal SYBR Green Supermix (Bio-Rad), 0.5 µL of both forward and reverse primers (final concentration of 10 µM), and 7 µL of ultrapure water, in a total reaction volume of 20 µL. The thermal cycling conditions for all targets, except for Firmicutes, were as follows: 95 °C for 3 min (1 cycle), followed by 95 °C for 10 s, 59 °C for 15 s, and 72 °C for 10 s (39 cycles), with a final step of 65 °C for 31 s, 65 °C for 5 s, followed by a 0.5 °C/cycle increase at a ramp rate of 0.5 °C/s (60 cycles).

For Firmicutes, the thermal protocol was adjusted to 95 °C for 2:30 min (1 cycle), followed by 95 °C for 10 s, 60 °C for 20 s, and 72 °C for 15 s (39 cycles), with a final step of 65 °C for 31 s, 65 °C for 5 s, followed by a 0.5 °C/cycle increase at a ramp rate of 0.5 °C/s (60 cycles).

The reaction efficiency ranged from 90% to 110%.

### 2.6. Data Analysis and Statistics

Statistical analyses were performed using the SPSS software package, version 27.0 (IBM Corp., Armonk, NY, USA). Descriptive analysis was conducted for all variables. Categorical variables were reported as absolute numbers and percentages, while continuous variables were expressed as means with standard deviations. The subjects were divided into two groups based on diagnosis: COVID-19 or MIS-C.

Differences between the COVID-19 and MIS-C groups were assessed using the χ² test with Fisher’s correction for categorical variables and the Mann–Whitney U test for independent samples for continuous variables. A *p*-value of <0.05 was considered statistically significant for all analyses.

For the biomolecular data, the following statistical approaches were applied: Log transformation was used for non-normally distributed data; Spearman’s correlation was applied to assess the relationships between variables; a *t*-test and paired *t*-test (where appropriate) were used to compare means; ANOVA was used for multivariate analysis, followed by a Tukey post hoc test for multiple comparisons. Mean differences and correlations were considered statistically significant for *p* < 0.05 and highly significant for *p* < 0.01.

For the microbiota analysis, data were processed using the microbiome R package. Alpha diversity was assessed using the following indices: Shannon, Simpson, Pielou, and Lladser indices [24]. Beta diversity was evaluated using several comparison models: Bray–Curtis, Jaccard, and Canberra/Manhattan.

For the microbiota, the data were analyzed using the microbiome R package software. For alpha diversity, the following indexes were calculated: Shannon, Simpson, Pielou, and Lladsser indexes. For the beta-diversity evaluation, different comparison models were applied: Bray–Curtis, Jaccard, and Camberra/Manhattan. Considering the potential influence of an exclusive milk diet on the gut microbiota, comparisons between the COVID-19 and MIS-C groups were performed and reported after excluding children under 6 months of age.

## 3. Results and Discussion

### 3.1. Cohort Characteristics and Clinical Treatments

As previously demonstrated in epidemiological studies, the MIS-C patients were older than those with COVID-19 in this study, as well (Table 1) [17]. This study was conducted at the primary pediatric hospital in Piedmont during the pandemic, where only the most severe COVID-19 cases were hospitalized. Infants are more vulnerable to severe clinical conditions following SARS-CoV-2 infections; consequently, 19% of the COVID-19 patients included in this study were under 6 months old, and 43% were under 1 year of age. No significant differences in ethnicity or gender distribution were observed within the cohort (Table 1).

Gastrointestinal symptoms were more frequent among the MIS-C patients despite a lower prevalence of SARS-CoV-2-positive stool samples (Table 1). This finding likely reflects, on the one hand, the greater involvement of the gastrointestinal system in MIS-C and, on the other hand, the reduced likelihood of detecting the virus in stool samples as more time elapses since the initial infection [25]. Nasopharyngeal swab positivity was universal in the COVID-19 cohort but only partially observed in the MIS-C patients (Table 1). Similar to stool samples, the probability of detecting the virus in biospecimens decreases as the time from the initial infection increases.

Pharmaceutical treatments for MIS-C patients predominantly included antibiotics and corticosteroids in most cases [17], which could independently impact the gut microbiota and subsequent outcomes, regardless of the SARS-CoV-2 infection. Other drugs were included sporadically and only for MIS-C patients under specific conditions (Table 1). Given the greater severity of MIS-C, the longer hospitalization duration observed in this study was expected (Table 1).

C-reactive protein (CRP) levels have been proposed in the literature as a biomarker for early prediction of COVID-19 infections [18,26,27], with CRP polymorphisms also linked to COVID-19 mortality [28]. In this study, the mean CRP levels observed in MIS-C patients were more than double those of children diagnosed with COVID-19, underscoring the importance of this inflammatory biomarker in assessing disease severity. Additionally, MIS-C patients experienced longer hospitalization durations than those with COVID-19, reflecting the higher severity of their clinical presentation. Gastrointestinal symptoms were also more frequent among MIS-C patients, indicating greater gut involvement in this condition.

The presence of SARS-CoV-2 RNA is higher in the nasopharyngeal tract than in stool samples among COVID-19 patients, which aligns with the shorter time elapsed since infection. The nasopharyngeal swabs tested positive in all COVID-19 patients but only in 38% of MIS-C patients. The estimated time since virus exposure was significantly longer for the MIS-C patients, with a mean of 31 days (range: 8–98 days), compared to a timeframe much closer to hospitalization for COVID-19 patients.

### 3.2. Alpha Diversity of the Microbiota

There was no significant difference in alpha diversity between MIS-C and COVID-19 patients (*p* > 0.05) (Table 2A). However, the Lladser index appeared to differ, suggesting the potential importance of low-abundance species in the gut microbiota [26]. The Shannon index values were lower than those typically observed in healthy children of comparable age (>3 years) [27], indicating reduced biodiversity and probable dysbiosis in both COVID-19 and MIS-C patients.

Alpha diversity was notably lower in younger COVID-19 patients (age < 0.5 years, Table 2B), which aligns with previous literature attributing these differences to the distinct microbiota of infants influenced by their feeding patterns. Despite these differences in diversity, the evenness appeared to be similar across samples, as indicated by the Pielou index [28]. A direct comparison of alpha diversity by age group for MIS-C patients was not possible due to the absence of patients under 0.5 years in this cohort (Table 2). This observation is consistent with surveillance data on MIS-C cases, which report a low prevalence of the disease in infants under 0.5 years of age (CDC, MIS-C Patients by Age Group, October 2023).

### 3.3. Beta-Diversity of the Microbiota

**Figure 1:**
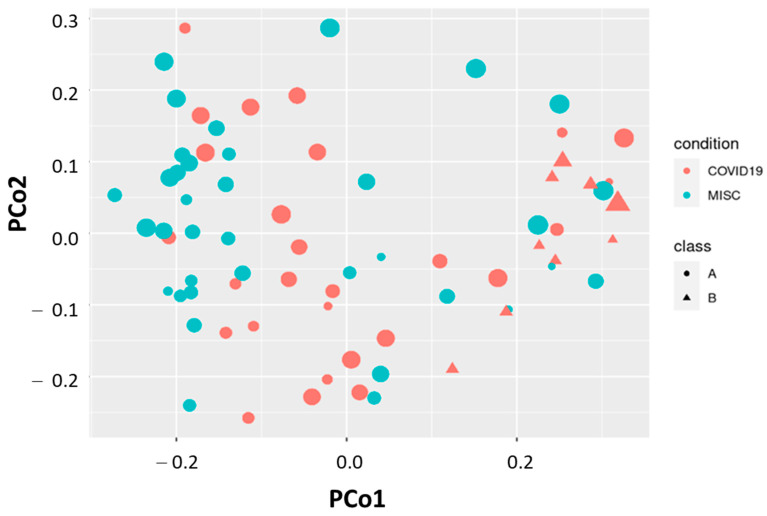
Principal coordinator analysis by Manhattan distance metric in relation to the patient condition (COVID-19 or MIS-C, red or blue color, respectively) and age class (cut off at 0.5 years, circle A—older; triangle B—younger).

Figure 1 presents the beta-diversity analysis plotted in relation to patient conditions (COVID-19 and MIS-C). A clear difference is evident when various approaches for the beta-diversity evaluation are applied, considering both age groups and patient conditions. When analyzing all patients, regardless of age group (nurslings, ≤6 months; older children, >6 months), the Adonis test using Jaccard, Bray–Curtis, and Manhattan indices consistently yielded significant results (*p* = 0.001). However, when nurslings were excluded, the Adonis test remained significant only for the Manhattan model (*p* = 0.021).

**Figure 2:**
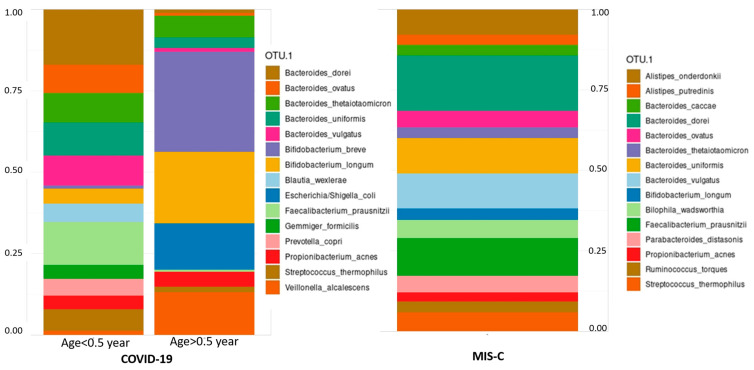
Relative abundance of main microbial species observed in COVID-19 (subdivided by age, cut off 0.5 years) and MIS-C.

As previously demonstrated, hospitalized COVID-19 patients were younger compared to MIS-C patients, and this age difference is associated with notable variations in both feeding practices and gut microbiota composition (Figure 2). The Manhattan metric, unlike the Bray–Curtis index, incorporates not only abundance data but also the absolute differences among coordinates. This emphasizes the contribution of low-abundance or marginal microbial genera to the observed dissimilarities [29].

### 3.4. Quantification of Specific Microbial Groups by qRT-PCR

The quantification of specific microbial groups, starting from DNA, is significantly and strongly correlated with evaluations based on mRNA (Table 3). This indicates that the presence of a microbial group (phylum, genus, or species, as determined by DNA) corresponds to its active form (as determined by mRNA under a transcriptomic approach). For instance, *Bacteroides* spp. shows a strong correlation with its phylum, Bacteroidetes, both in terms of presence and activity. In contrast, for Firmicutes, a reduced correlation (rho) between presence and active form may be attributed to the presence of “silent” microbes, which are detectable by DNA but exhibit limited transcriptional activity.

*Bifidobacterium* spp. (from the phylum Actinobacteria) is significantly correlated with the presence of *Akkermansia muciniphila* (from the Verrucomicrobia phylum). However, the activity levels of these two microbial groups do not correlate with each other or with other quantified microbial groups. This finding highlights the potential importance of low-abundance species in the gut when defining microbiota signatures associated with disease. Additionally, *Bifidobacterium* spp. (from the phylum Actinobacteria) is significantly correlated with the presence of *Akkermansia muciniphila* (from the Verrucomicrobia phylum). However, the activity levels of these two microbial groups do not correlate with each other or with other quantified groups. This observation reinforces the importance of low-abundance species in the gut when identifying microbiota signatures associated with disease.

Quantifications of specific microbial groups based on DNA are significantly and strongly correlated with those derived from mRNA evaluations (Table 3). This suggests that the presence of a microbial group (phylum, genus, or species) detected through DNA is generally matched by its active form, as assessed through mRNA under a transcriptomic approach.

For *Bacteroides* spp., a strong correlation was observed with its phylum, Bacteroidetes, in terms of both presence and activity. In contrast, a reduced correlation (rho) between the presence and activity of Firmicutes may indicate the existence of “silent” microbes—microbial groups detectable via DNA but with limited transcriptional activity.

The significant correlation between the presence of *Bifidobacterium* spp. (Actinobacteria) and *Akkermansia muciniphila* (Verrucomicrobia) further supports the importance of microbial interactions. However, the activity levels of these two microbial groups are not correlated with each other or with other quantified groups. This discrepancy emphasizes the role of low-abundance species in the gut, suggesting they may contribute significantly to microbiota-disease signatures, even when their activity levels are less apparent.

**Figure 3:**
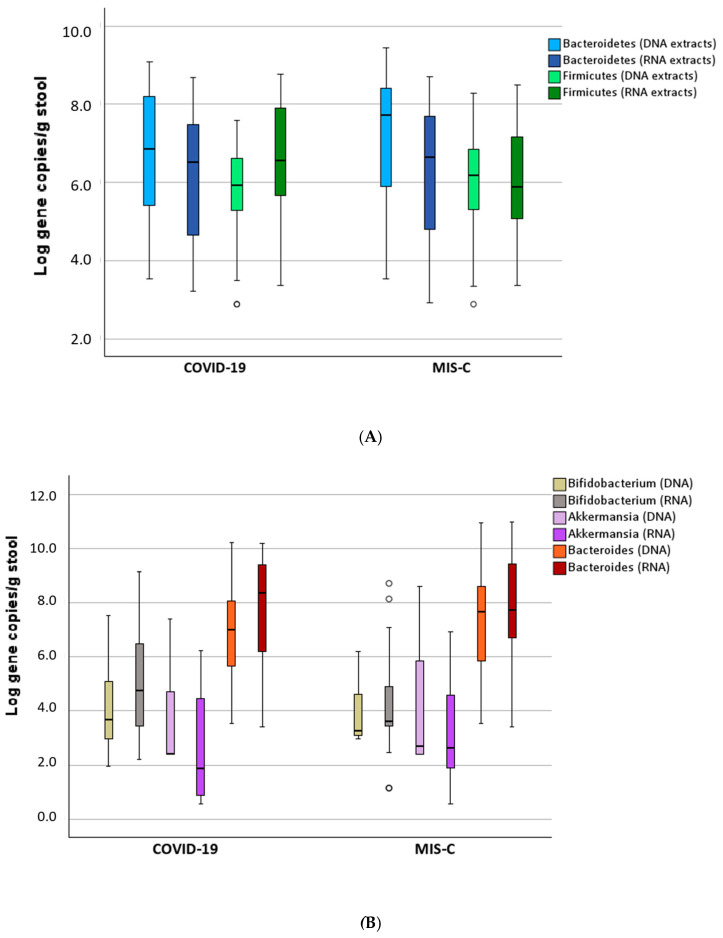
Box plot of the qRT-PCR results subdivided by disease, starting both from DNA and mRNA extracts: for Phyla (**A**) and for detected genera and species (**B**). The white dots represent the outliers.

The composition in terms of phyla and more specific microbial targets is shown in Figure 3A and Figure 3B, respectively. The similar abundance observed by NGS methods between COVID-19 and MIS-C, both for DNA and mRNA, was further confirmed using the qRT-PCR method with degenerate primers.

The age group <0.5 years influenced both the presence of Bacteroidetes and Firmicutes (*p* < 0.05) but not the specific genera or species. These findings were corroborated by the NGS microbiome analysis, where both Bacteroidetes and Firmicutes showed an increase over the lifespan (Spearman’s rho = [value] and *p* < 0.05, respectively).

Among the specific genera, Bifidobacterium was one of the most variable microbial targets. It differed between COVID-19 and MIS-C patients, both when the RNA was analyzed (5.14 vs. 4.21 Log gene copies/mg feces, *p* < 0.05) and between SARS-CoV-2-positive and negative stool samples for both DNA and RNA analyses (2 Log higher concentration in the positive stools, *p* < 0.05). These results were influenced by the higher proportion of very young infants with COVID-19 and SARS-CoV-2-positive stool. Furthermore, *Bifidobacterium* levels were affected by antibiotic treatment, showing a decrease of 29% (*p* = 0.002).

The C-reactive protein (CRP) level increased with the levels of *Akkermansia muciniphila* (Spearman’s rho = 0.265, *p* = 0.043), although this bacterium was generally present at low levels (30% of samples were below the limit of quantification, LOQ). An impact of sporadic pharmaceutical treatments was observed. Specifically, cortisone inhibited both the presence and expression of Bacteroidetes and other microbes, and more specifically, *Bacteroides* spp., *Bifidobacterium* spp., and *Akkermansia muciniphila* (*p* < 0.05).

## 4. Conclusions

The incidence of MIS-C is much lower after the pandemic; however, it may still occur during or following periods of increased SARS-CoV-2 circulation. A correct diagnosis of MIS-C is crucial for appropriate treatment, monitoring trends, and understanding patients’ demographic and clinical characteristics. Recent MIS-C cases have been observed in unvaccinated children, underscoring the importance of COVID-19 vaccination as a tool for preventing MIS-C.

Recruiting patients and biological samples is challenging but possible, even in emergency situations. Factors such as young age and treatments with antibiotics and cortisone significantly affect the microbiota, which is an important confounding factor to consider when planning sample collection for diseased children. The gut microbiome plays a role in the adverse effects of SARS-CoV-2 infection, though this role is part of a complex, multisystemic response of the child’s body.

Weak differences in gut microbiota profiles were observed at the time of hospitalization between children with COVID-19 and those with MIS-C, particularly concerning low-abundance microbial species in the gut, based on both alpha- and beta-diversity analyses. *Bacteroides* spp., *Bifidobacterium* spp., and *Akkermansia muciniphila* are key components of the microbiota and are often proposed as biomarkers of gut health. However, in this context, they do not serve as proxies for MIS-C progression.

An in-depth evaluation of other genera and species, especially when associated with immunological biomarkers, could be useful for developing treatments that reduce the risk of severe outcomes. Additionally, such evaluations could help establish microbiota-based biomarkers for the early diagnosis, treatment, and subsequent restoration of MIS-C.

## Figures and Tables

**Table 1:** The cohort includes data on the number of patients, sex, age, and clinical variables. Continuous variables are presented as means with standard deviations, while categorical variables are expressed as percentages. Statistical comparisons between MIS-C and COVID-19 groups include corresponding *p*-values to highlight significant differences.

		COVID-19 (Mean ± SD or Percentage)	MIS-C (Mean ± SD or Percentage)	*p*-Value (Test χ^2^ or Mann–Whitney)
Age (years)		5.1 ± 4.9	8.1 ± 4.0	0.004
Sex	F	56.8%	59.5%	=0.814
	M	43.2%	40.5%	
Gastrointestinal symptoms	No	67.6%	19.4%	<0.001
	Yes	32.4%	80.6%	
Antibiotics	No	45.9%	2.8%	<0.001
	Yes	54.1%	97.2%	
Cortisone	No	94.6%	69.4%	0.005
	Yes	5.4%	30.6%	
Immunoglobulins	No	100.0%	91.7%	0.073
	Yes	0.0%	8.3%	
Antimycotics	No	100.0%	97.2%	0.307
	Yes	0.0%	2.8%	
Hospitalization days		8.7 ± 7.1	12.2 ± 3.8	<0.001
Days since nasopharyngeal swab		8.9 ± 15.2	13.3 ± 16.1	0.125
CRP		85.7 ± 226.2	201.0 ± 99.6	<0.001
DNA ng/μL		22.64 ± 18.48	34.31 ± 31.68	0.155
RNA ng/μL		104.45 ± 152.29	54.86 ± 74.82	0.118
SARS-CoV-2 RNA in stool	No	64.9%	91.9%	0.005
	Yes	35.1%	8.1%	
Nasopharyngeal swab positivity	No	0%	62%	<0.001
	Yes	100%	38%	

**Table 2:** Alpha-diversity patients expressed as mean and standard deviation of the following indexes: Shannon, Simpson, Pielou, and Lladser.

**(A) COVID-19 and MIS-C and *p*-Value of the Wilcoxon’s Test**			
**Alpha Diversity** **Index**	**COVID-19** **(Mean ± s.d.)**	**MIS-C** **(Mean ± s.d.)**	***p*** **-Value**
Shannon	2.641 ± 0.809	2.889 ± 0.770	0.2285
Simpson	0.705 ± 0.166	0.754 ± 0.133	0.2789
Pielou	0.606 ± 0.152	0.618 ± 0.111	0.9606
Lladser	0.003 ± 0.008	0.016 ± 0.003	0.0248
**(B) Only COVID-19 Children Comparing by Age and *p*-Value of the Wilcoxon’s Test**			
**Alpha Diversity** **Index**	**Age ≥ 0.5 Year (N. 27)** **(Mean ± s.d.)**	**Age < 0.5 Year (N. 9)** **(Mean ± s.d.)**	***p*** **-Value**
Shannon	2.8342 ± 0.7382	2.062 ± 0.768	0.00873
Simpson	0.7387 ± 0.1551	0.60548 ± 0.1674	0.03919
Pielou	0.622 ± 0.147	0.557 ± 0.164	0.2626
Lladser	0.0013 ± 0.0008	0.0087 ± 0.0159	0.00873

**Table 3:** Correlation among the concentrations of the microbials detected by qRT-PCR starting from DNA (yellow) and from mRNA (Blue). Spearman’s rho and its significance (* or **) are included for each couple, followed by the *p*-value for each line.

	*Bacteroidetes*	*Firmicutes*	*Bacteroides*	*Bifidobacterium*	*Akkermansia*	*Bacteroidetes*	*Firmicutes*	*Bacteroides*	*Bifidobacterium*	*Akkermansia*
***Bacteroidetes***	1.000	0.369 **	0.900 **	0.083	0.087	0.657 **	0.195	0.513 **	0.162	−0.067
		0.001	0.000	0.487	0.470	0.000	0.099	0.000	0.171	0.571
***Firmicutes***		1.000	0.332 **	0.226	0.252 *	0.275 *	0.302 **	0.275 *	0.018	0.157
			0.004	0.054	0.033	0.019	0.009	0.019	0.880	0.185
***Bacteroides***			1.000	−0.006	0.142	0.699 **	0.245 *	0.480 **	0.117	−0.145
				0.957	0.235	0.000	0.036	0.000	0.323	0.221
***Bifidobacterium***				1.000	0.281 *	−0.035	−0.010	−0.057	0.332 **	0.037
					0.017	0.770	0.936	0.633	0.004	0.758
***Akkermansia***					1.000	0.298 *	0.142	0.052	0.145	0.496 **
						0.011	0.236	0.666	0.225	0.000
***Bacteroidetes***						1.000	0.597 **	0.768 **	0.417 **	0.180
							0.000	0.000	0.000	0.124
***Firmicutes***							1.000	0.686 **	0.454 **	0.188
								0.000	0.000	0.109
***Bacteroides***								1.000	0.442 **	0.107
									0.000	0.368
***Bifidobacterium***									1.000	0.155
										0.188

## Data Availability

The data presented in this study regard children and are available upon request from the corresponding author due to ethical reasons in the observation of privacy regulations.

## Supplementary Materials

The following supporting information can be downloaded at: https://www.mdpi.com/article/10.3390/microorganisms13010083/s1.

## Abbreviations

COVID-19	CoronaVirus Disease 19
SARS-CoV-2	Severe Acute Respiratory Syndrome CoronaVirus 2
RNA	Ribonucleic Acid
mRNA	Messenger Ribonucleic Acid
DNA	Deoxyribonucleic Acid
cDNA	Complementary Deoxyribonucleic Acid
ACE2	Angiotensin-Converting Enzyme 2
MIS	Multisystem Inflammatory Syndrome. It can affect children (MIS-C) or adults (MIS-A)
ELISA	Enzyme-linked Immuno-Sorbent Assay
DIN	DNA Integrity Number
RNP	RiboNucleoProtein
PCR	Polymerase Chain Reaction
RT-PCR	Reverse Transcription Polymerase Chain Reaction
qRT-PCR	Quantitative Real-Time Polymerase Chain Reaction
NGS	Next-Generation Sequencing
CRP	C-Reactive Protein
