# Supplementary material for: Investigating the Role of Gut Microbiota in Pediatric Patients with Severe COVID-19 or MIS-C
Source: Microorganisms. 2025 Jan 4;13(1):83. doi: 10.3390/microorganisms13010083 (PMC11767266; doi:10.3390/microorganisms13010083)
Supplement: Supplementary file 1 [file microorganisms-13-00083-s001.zip › microorganisms-3377530-supplementary.pdf]

Dipartimento
Scienze della Sanità
Pubblica e Pediatriche

UNIVERSITÀ
DI TORINO

AZIENDA OSPEDALIERO - UNIVERSITARIA
Città della Salute e della Scienza di Torino

DESCRIPTION OF THE VERBAL INFORMED CONSENT

Title of the Study:

Investigating the Role of Gut Microbiota in Pediatric Patients with Severe COVID-19 or MIS-C

Principal Investigator: Prof.ssa Deborah Traversi, dott.ssa Emilia Parodi
Institution: University of the Study of Turin, Department of Public Health and Pediatrics
Contact Information: +390116705703, deborah.traversi@unito.it

Introduction
You are being invited to participate in a research study investigating stool samples from children diagnosed with Multisystem Inflammatory Syndrome in Children (MIS-C)/or COVID-19 (in relation to the different diagnoses). This study aims to better understand the effects of these conditions on the gastrointestinal system. Participation is entirely voluntary, and you may withdraw at any time without any impact on your medical care.

Purpose of the Study
The purpose of this study is to analyse stool samples to identify potential biomarkers or microbial changes associated with MIS-C or COVID-19. This research could help improve diagnosis and treatment in the future.

What Participation Involves
If you agree to participate:

1. You will provide a stool sample from your child for analysis.
2. Additional information about your child's medical history and current health may be collected through a questionnaire or medical records, with your consent.
3. The sample and data will be anonymized to protect your child's identity.

Duration of Participation
Participation will involve a one-time stool sample collection. There are no follow-up requirements unless additional clarification is needed.

Risks and Benefits

- **Risks:** There are no risks associated with participation. Stool sample collection is totally non-invasive.
- **Benefits:** While there may be no direct benefit to your child, the study may contribute to scientific knowledge that improves the care of children with MIS-C or COVID-19.

Confidentiality
All personal information will be kept strictly confidential. Samples and data will be assigned a unique code,

and only the research team will have access to the code key. Results will be reported in aggregate form, ensuring that no individual can be identified.

Voluntary Participation and Withdrawal
Participation is entirely voluntary. You can choose not to participate or withdraw your consent at any time without affecting your child's medical care.

Contact Information for Questions or Concerns
If you have any questions about this study or your rights as a participant, please contact:
Dott.ssa Emilia Parodi, emilia.parodi@unito.it

Verbal Consent Statement
I have listened and understood the information provided above. I have had the opportunity to ask questions and have them answered. I voluntarily agree to allow my child to participate in this study.

As consequence of the verbal consent an annotation was reported on the patient medical record by the attending physician
